# NEMoE: a nutrition aware regularized mixture of experts model to identify heterogeneous diet-microbiome-host health interactions

**DOI:** 10.1186/s40168-023-01475-4

**Published:** 2023-03-15

**Authors:** Xiangnan Xu, Michal Lubomski, Andrew J. Holmes, Carolyn M. Sue, Ryan L. Davis, Samuel Muller, Jean Y. H. Yang

**Affiliations:** 1 Charles Perkins Centre, The University of Sydney, Camperdown, Sydney, NSW Australia; 2 School of Mathematics and Statistics, The University of Sydney, Camperdown, Sydney, NSW Australia; 3 Department of Neurology, Royal North Shore Hospital, Northern Sydney Local Health District, St Leonards, NSW Australia; 4 Department of Neurogenetics, Kolling Institute, Faculty of Medicine and Health, University of Sydney and Northern Sydney Local Health District, St Leonards, NSW Australia; 5 The University of Notre Dame Australia, School of Medicine, Sydney, NSW Australia; 6 School of Life and Environmental Sciences, University of Sydney, Camperdown, Sydney, NSW Australia; 7 Department of Mathematics and Statistics, Macquarie University, Sydney, NSW 2109 Australia; 8 Laboratory of Data Discovery for Health Limited (D24H), Science Park, Hong Kong, SAR China

**Keywords:** Latent class, Subcohort, Microbiome, Nutrition, Mixture of experts

## Abstract

**Background:**

Unrevealing the interplay between diet, the microbiome, and the health state could enable the design of personalized intervention strategies and improve the health and well-being of individuals. A common approach to this is to divide the study population into smaller cohorts based on dietary preferences in the hope of identifying specific microbial signatures. However, classification of patients based solely on diet is unlikely to reflect the microbiome-host health relationship or the taxonomic microbiome makeup.

**Results:**

We present a novel approach, the Nutrition-Ecotype Mixture of Experts (NEMoE) model, for establishing associations between gut microbiota and health state that accounts for diet-specific cohort variability using a regularized mixture of experts model framework with an integrated parameter sharing strategy to ensure data-driven diet-cohort identification consistency across taxonomic levels. The success of our approach was demonstrated through a series of simulation studies, in which NEMoE showed robustness with regard to parameter selection and varying degrees of data heterogeneity. Further application to real-world microbiome data from a Parkinson’s disease cohort revealed that NEMoE is capable of not only improving predictive performance for Parkinson’s Disease but also for identifying diet-specific microbial signatures of disease.

**Conclusion:**

In summary, NEMoE can be used to uncover diet-specific relationships between nutritional-ecotype and patient health and to contextualize precision nutrition for different diseases.

Video Abstract

**Supplementary Information:**

The online version contains supplementary material available at 10.1186/s40168-023-01475-4.

## Background

The human body is home to complex microbial communities, collectively known as the microbiome, which is mostly made up of prokaryotes (bacteria) and archaea [1]. Considerable evidence has emerged indicating that the microbiome is an important contributor to an individual’s health [2]. This has been illustrated by links between the gut microbiome and numerous diseases, including irritable bowel syndrome [3], Crohn’s disease [4], type 2 diabetes [5], cardiovascular disease [6], and Parkinson’s disease (PD) [7]. The gut microbiome is known to change throughout our lives as a result of various environmental influences. Diet, being one of these factors, has the greatest known long-term interaction with the gut microbiome [8]. Thus, a deep understanding of the relationship between diet and the gut microbiome and the consequential impact on disease processes holds promise for developing personalized dietary intervention strategies to modulate and maintain a healthy microbiome population [9, 10].

**Fig. 1:**
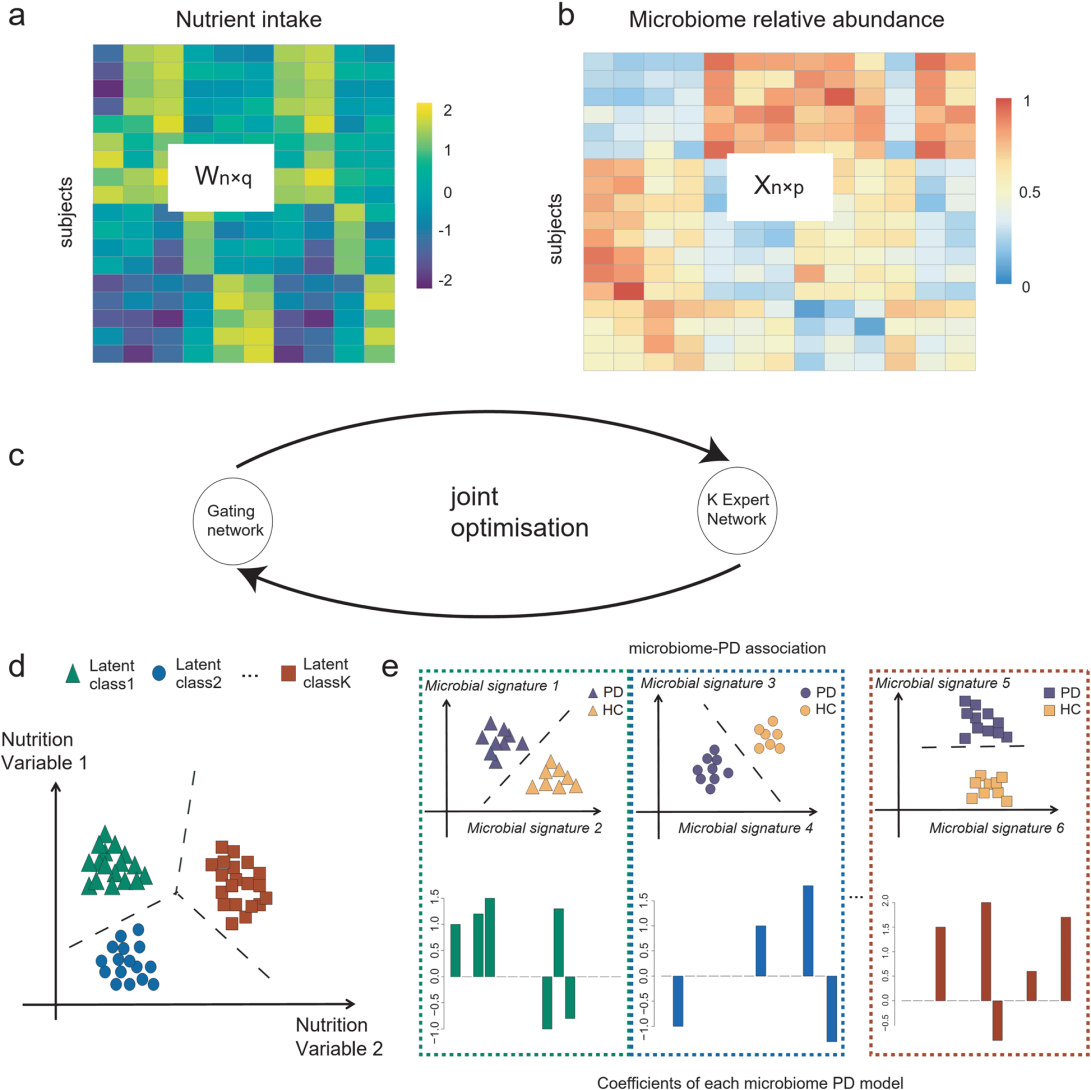
Illustration of NEMoE: **a**, **b** The input matrix of NEMoE: *n* samples with *q* nutrient features and *p* microbial features. **c** A conceptual workflow of NEMoE, where the joint optimization is achieved by EM algorithm to maximize the regularized likelihood function. **d** A toy example showing a nutritional-ecotype in the microbiome PD study. The nutrient intake is clustered into *K* latent classes. **e** In each latent class, the microbial signatures of PD are different, which is reflected by the coefficients in the experts network

Diet has a direct impact on the microbial community in the gut, which governs the activity of the intestinal ecosystem and can have considerable implications for an individual’s health [11, 12]. This is conceptualized in Fig. 1 where, for illustration purposes, the macronutrient intake is separated into three perfectly distinct subcohorts with different associations between microbiome composition and PD. In practice, several studies have demonstrated that variations in nutrient intake, such as different ratios of protein, carbohydrate [13], or dietary fiber [14] intake, can influence the host-microbiome association. These discoveries are generally based on an elaborate experimental design using model organisms [13] or dietary interventions [15–17]. Recent observational studies suggest that long-term diets could be associated with the microbiome [18], and this can further affect overall health. In a similar context, our recent study of the gut microbiome in PD showed that when partitioning individuals based on carbohydrate intake, the predictive performance of the microbiota profile to indicate PD was increased [19, 20]. Together, these studies suggest that dietary differences can impact relationships between microbiome composition and host health/disease status.

To uncover complex heterogeneous relationship structure between diet, microbiome, and host health, it is important to identify homogeneous subcohort or latent structure in data that can be explained by a set of features. This is similar to the concept of “ecotype”, which is commonly used to refer to a variant which has observable phenotypically difference in a local environment [21]. Hence, using a data-driven approach, it is able to divide a population into multiple subcohorts with distinct microbiological signatures for health that can be best described by nutrient combinations, resulting in what we term “nutritional-ecotypes.” These subcohorts can be thought of as diet-based latent classes where they capture interaction between the constraints imposed by nutrient intake of individuals on the community dynamics of their microbiomes [22, 23].

Methods to discover such diet-based latent classes could be hypothesis-driven based on prior knowledge [24, 25] or guided by an unsupervised statistical learning method, such as clustering [26], followed by latent class analysis [27]. Although these methods identify nutrient-classes with an altered overall nutritional profile, one limitation is that the defined cohorts may not reflect the heterogeneous microbiome-host health relationship: the drivers of “diet x microbiome” outcomes, “diet x host” outcomes, and “host x microbiome outcomes” are overlapping, but not perfectly congruent. Consequently, classification models built within a subcohort defined just by diet (or microbiome) will not necessarily improve prediction of the health/disease state [28].

Similar concepts of identifying cohort heterogeneity to improve prediction performance have been developed in other omics settings and for other diseases [29, 30]. However, simple adaptations of methodologies developed for other omics platforms remain challenging as these do not account for the hierarchical taxonomic structure observed in the study of the diet-microbiome-host interaction. That is, each individual should be in the same diet-specific cohort across all taxonomic levels to keep hierarchical fidelity of the microbial community, i.e., a consistent nutrition class across Phylum, Class, Family, Genus, etc.

To this end, we propose a novel *N*utritional-*E*cotype *M*ixture *o*f *E*xperts (NEMoE) approach for uncovering associations between the gut microbiome profile and the health state of an individual that takes into account diet-specific cohort heterogeneity (Fig. 1 and Supplementary Fig. 1 and 2). This is achieved by using a regularized mixture of experts model to simultaneously optimize the separations between nutritional-ecotypes, classification performance of microbiota, and the health state. The mixture of experts models has been widely used in integrating different types of data. Kim and colleagues [31] have used it for combining clinical data and genomics data. However, this work does not use sparse regularization and lacks interpretability, i.e., unable to identify unique markers in each experts network. NEMoE also integrates a model parameter sharing strategy to account for the taxonomic information contained in microbiome data, ensuring coherent nutritional classification is maintained across all taxonomic levels. We show through empirical computational simulation research that NEMoE is robust to parameter changes. We also apply NEMoE to real microbiome data from a PD cohort and show that the model outperforms existing approaches of predictive performance and is able to uncover candidate diet-specific microbiome markers of complex disease.

## Results

### NEMoE, a novel method for jointly identifying nutritional-ecotype and for modeling the relationship between microbiota and health state

NEMoE identifies nutritional-ecotypes that represent differential dietary intake as well as the relationship between microbiome structure and host health (Fig. 1 and Supplementary Fig. 1). This approach has two distinct components: first, a gating network aimed at estimating latent classes shaped by nutritional intake, and second, an experts network aimed at modeling the relationship between the microbiota composition and the health state within each latent class [31, 32]. The input of the gating network is a nutrition matrix, with each variable being the nutrients intake of the individual and the corresponding microbiome measurements are used as input of the experts network. Similar to non-regularized mixture of experts (MoE) models, fitting NEMoE involves estimating the parameters via maximum likelihood estimation to simultaneously optimize the separations among nutritional-ecotypes, microbiome classification performance, and the health state (Supplementary Fig. 2). The optimization procedure is usually achieved by an expectation maximization (EM) algorithm. However, the MoE model does not extend to a large number of feature variables (*p*) and small sample size (*n*) framework, which often occurs in diet and microbiome data where there are many more features than observations. Instead, NEMoE adopts a regularization component to the MoE (RMoE [33]) by adding elastic net penalties [34] on both the gating function and the experts network (details in the Methods section). Next, NEMoE employs a parameter sharing strategy that involves a shared gating network for the microbiome relative abundance matrices across taxonomic levels, to ensure coherent latent classes across all taxonomic levels. Compared with a latent class using purely nutritional intake, our nutritional-ecotype has two advantages: (i) it takes the relationship between microbiome and health outcome into account and is beneficial for identifying diet-specific microbial signatures (Supplementary Fig. 1). (ii) It incorporates the taxonomic structure in the latent class and keeps hierarchical fidelity of the microbial community.

### NEMoE is able to accurately identify nutritional latent classes shared across different taxonomic levels

**Fig. 2:**
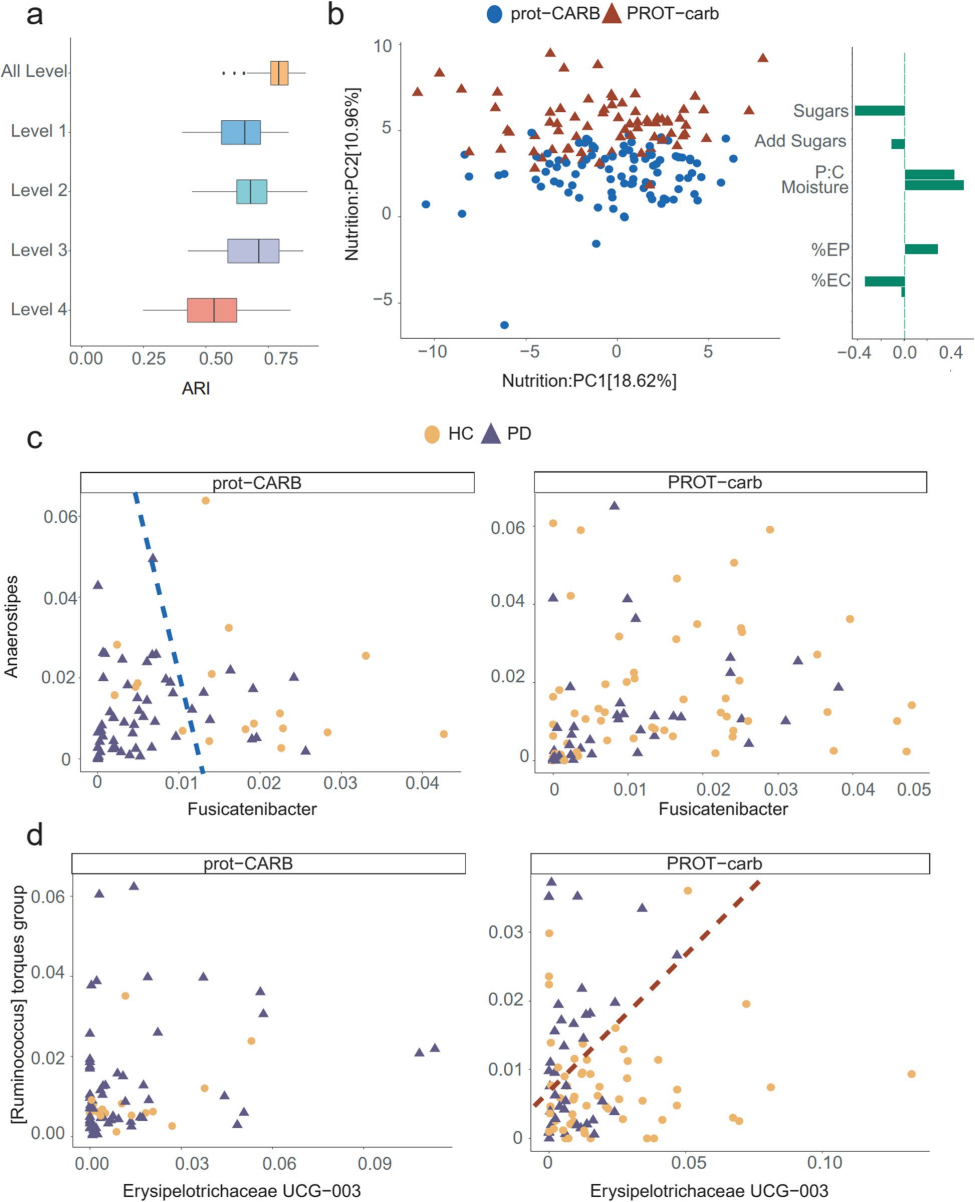
Identification of nutritional-ecotype by NEMoE. **a** Boxplot comparing NEMoE and single-level NEMoE in estimating shared latent classes. The ARI (*x*-axis) is calculated by comparing the estimated latent class and the true latent class from the data-generating model. In all settings, NEMoE using multiple-level information performs better. **b** PCA plot of scaled nutrient intake for subjects colored by the two nutrition classes as estimated by NEMoE. Estimated coefficients of the gating network showed high coefficients for sugar, protein:carbohydrate, and moisture. We denote the two nutrition classes as prot-CARB and PROT-carb with low protein-high carbohydrate intake and vice versa. **c** Scatter plot of genera *Fusicatenibacter* and *Anaerostipes*. Left panel shows that Parkinson’s disease and healthy controls in the prot-CARB subcohort roughly separate but there is no such separation in the PROT-carb right panel. **d** Scatter plot of genera *Erysipelotrichaceae UCG-003* and *[Ruminococcus] torques group* showed a different relationship between Parkinson’s Disease and Healthy Controls in two nutritional-ecotypes

We evaluated the efficiency of NEMoE in determining nutritional-ecotypes based on microbiota across different taxonomic levels using both simulated and experimental data. In our simulation study (see Supplementary Notes), we created a four-level dataset of 500 samples with shared latent structure, where each individual belonged to a nutritional-ecotype and the relationship between microbiota and health status differed between two simulated nutritional-ecotypes. The adjusted rand index (ARI), a cluster comparison statistic, was used to compare the estimated nutritional-ecotypes and the underlying simulated latent classes (Fig. 2a). We discovered that by incorporating hierarchical taxonomy information in our NEMoE approach, the estimated nutritional class was cohesive and performed better (higher ARI = 0.80) than nutritional-ecotypes estimated from a single taxonomy level (ARI = 0.75). NEMoE achieved this by sharing information across taxonomic levels and the estimated latent class incorporated information from all levels.

Next, we applied NEMoE to our in-house data from a gut microbiome PD study . A scatter plot from the first two components of a principal component analysis (PCA) of scaled nutrient intake (see Methods section) from all individuals is shown in Fig. 2b, with the two nutritional-ecotypes best described as “high protein”–“low carbohydrate” (PROT-carb; shown in red) and “low protein”–“high carbohydrate” (prot-CARB; blue). The corresponding loadings show that these two ecotypes have very different ratios of protein to carbohydrate intake: Sugars and %EC (percentage of energy intake as carbohydrate) showed negative coefficient (γ < 0); P:C, Moisture and %EP (percentage of energy intake as protein) showed a positive coefficient of the gating network (γ > 0). Based on the meaning of these variables, we described the groups as “PROT-carb” and “prot-CARB,” with capital letters indicating the variable with a positive coefficient. Figure 2c and d illustrate that the relationships between gut microbiota and PD status are different between these two nutrition-ecotypes, PROT-carb, and prot-CARB. It is important to note that two identified subcohorts are significantly different to clusters identified by unsupervised clustering, such as subcohorts estimated by the *k*-means algorithm (ARI ~ 0, Supplementary Fig. 3).

**Fig. 3:**
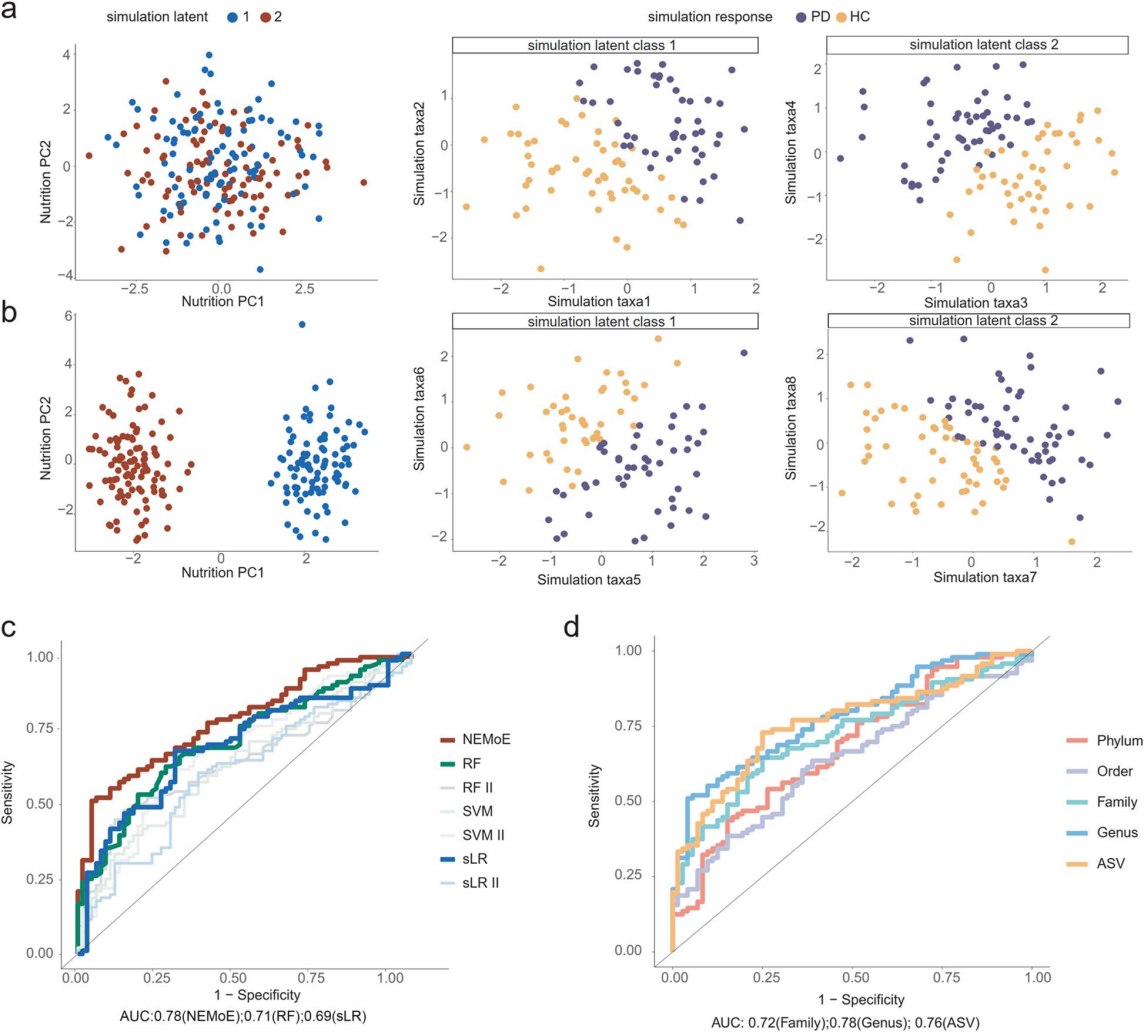
Comparison of NEMoE on simulation dataset and real dataset. **a** An illustration of a non-separable case where nutrition intake does not show a difference between two nutritional-ecotypes, but each subcohort shows a different relationship between microbiome taxa and health state. **b** An illustration of a separable case where nutrition intake is significantly different between two nutritional-ecotypes and relationships in each model are similar to the illustration in **a**. Simulation studies showed that NEMoE can identify both case **a** and case **b**. **c** Receiver operating characteristics curve of different methods (see Table 1) in predicting Parkinson’s disease using LOOCV. NEMoE showed the best LOOCV-AUC (AUC = 0.78). **d** ROC plot of NEMoE at different taxonomic levels using LOOCV. Genus level showed the best predictive performance (AUC = 0.78)

We further established the generalizability of NEMoE by examining its impact when applied to data with different levels of heterogeneity. Here, we created synthetic datasets with four different degrees of separation (Fig. 3a, b and Supplementary Notes) and demonstrated that NEMoE performs better than other existing approaches in detecting latent classes and this difference was more evident in challenging situations where the true separation between latent classes was small (Supplementary Fig. 4). This implies that NEMoE has potential to perform well in many observational studies where nutrient intake patterns are mixed or difficult to separate, and hence the NEMoE approach can be applied broadly to human disease datasets with diverse dietary intake.

### NEMoE outperforms existing supervised methods in predicting Parkinson’s disease state

We evaluated the predictive performance of NEMoE using both simulation and real data based on leave-one-out cross-validation (LOOCV; see Supplementary Notes) to the area under the receiver operating characteristics curve (AUC) for the various models described in Table 1. In simulation studies, we showed that under all comparison settings, NEMoE was able to achieve higher prediction accuracy (Supplementary Fig. 4), which implies NEMoE is robust to different parameter settings, such as *n* and *p*. Figure 3c highlights that when NEMoE was applied to our in-house dataset from a gut microbiome PD study [20] with 2 latent classes (AUC = 0.78), it outperformed all other approaches, with the next best being random forest (AUC = 0.71). Supplementary Fig. 6 further highlights that increasing the number of latent classes for this data did not improve the overall AUC.

**Table 1:** Summary of methods for comparison

Method	Input data of identified subcohort	Input data of modeling within each subcohort	Model
sLR		Microbiome	Sparse logistic regression
SVM		Microbiome	Support vector machine
RF		Microbiome	Random forest
sLR K^a^	Nutrition	Microbiome	Two-stage sLR with K latent class
SVM II	Nutrition	Microbiome	Two-stage SVM
RF II	Nutrition	Microbiome	Two-stage RF
NEMoE K^b^	Nutrition	Microbiome	NEMoE with K latent class
MMMoE^c^	Microbiome	Microbiome	RMoE
NNMoE	Nutrition	Nutrition	RMoE
MNMoE	Microbiome	Nutrition	RMoE
Comb-MoE	Microbiome+nutrition	Microbiome+nutrition	RMoE

^a^Two stage sparse logistic regression fitted with two, three four latent classes were denoted as sLR II, sLR III, and sLR IV

^b^NEMoE fitted with two, three four latent classes were denoted as NEMoE II, NEMoE III, and NEMoE IV. When not explicitly including the number of latent classes, we refer to NEMoE II

^c^Our NEMoE is easy to extend to partition the population with different types of data. We also investigate the different types of data as input of the NEMoE model. Results showed using nutrition to split the population obtained the best performance in our dataset

NEMoE’s ability to detect meaningful subcohorts via its joint optimization approach is a key driver of this increase in accuracy. For example, when comparing to a naive two-stage model that uses unsupervised clustering to identify latent classes before fitting two independent models, the performance of NEMoE is considerably better, as indicated by the large difference in AUC (NEMoE = 0.78, sLR II= 0.6). We further assessed NEMoE’s capabilities on enterotype-separated subcohorts [35] within our PD dataset. Enterotype, a widely used concept in microbiome research, refers to the categorization of an individual’s microbiomes by the variance in composition [2, 36]. It is widely accepted that enterotype captures stable compositional features of individuals and differences in community-type prevalence across populations with different long-term diets. In this study, we classify 87 samples as Enterotype B, 81 samples as Enterotype F, and no samples as Enterotype P. The cluster memberships between the subcohorts determined by NEMoE and by enterotype had no more overlap than pure chance (ARI = 0). Furthermore, building a different classifier for each of the two enterotypes had a much lower (LOOCV-AUC = 0.65) predictive ability than NEMoE (LOOCV-AUC = 0.78). This suggests that NEMoE allows the model to focus more on each latent class and increases prediction performance by more precisely identifying subcohorts with differential microbiome-PD relationships.

### Identification of informative taxonomic levels and consensus candidate microbial PD signatures in multiple independent cohorts

In our in-house gut microbiome PD investigation, NEMoE provided a natural criterion to examine which of the taxonomic levels (Phylum, Order, Family, Genus, and ASV) was most informative with respect to different nutrient intakes. We achieved this by evaluating predictive performance for PD at each taxonomic level to determine the most informative. Figure 3c shows that genus was most predictive compared to the other taxonomic levels, with an LOOCV-AUC of 0.78.

**Fig. 4:**
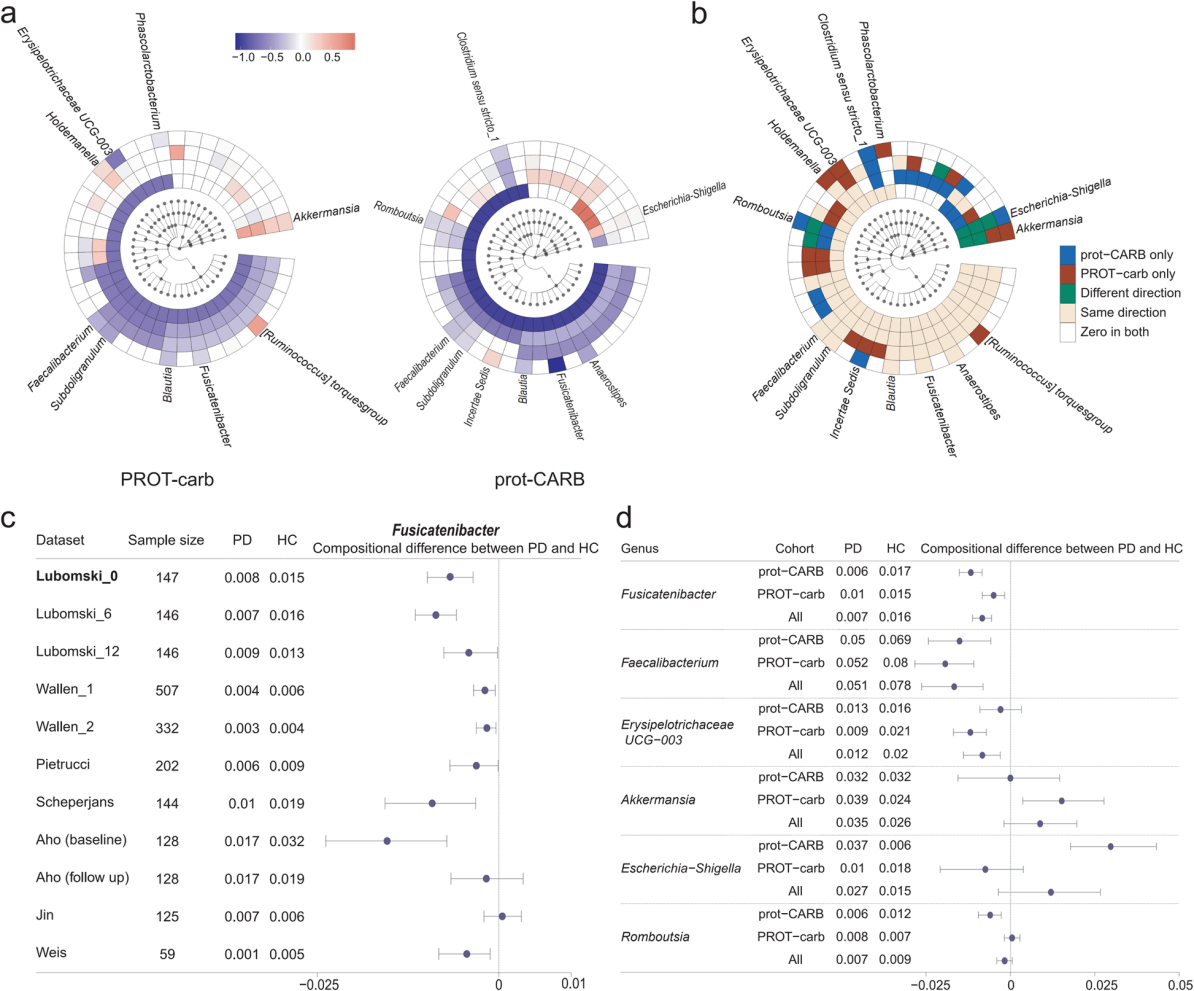
Results of NEMoE on gut microbiome-PD study. **a** Coefficients of experts network in NEMoE at different taxonomic levels. The two latent classes showed distinctly different microbiome patterns. **b** Identification of diet-specific microbial signatures of PD. The “Same direction*”* class showed consistent function in different dietary patterns. The “PROT-carb only” and “prot-CARB only” classes tended to be important only with specific dietary intake. The “Different direction” class changed their coefficients in different dietary patterns. **c** Validation of differential relative abundance of genus *Fusicatenibacter* in 11 different datasets. With the exception of one dataset (Jin et al. [37]) all other datasets showed decreasing *Fusicatenibacter* in PD. **d** Forest plot of 95% confidence interval of selected taxa showed NEMoE is able to identify the species that are differentially represented in specific nutritional-ecotypes

Next, our NEMoE model determined a separate set of PD microbial signatures for each nutritional-ecotype. The derived coefficients represent the level of association between microbiota and health/disease state in each nutritional-ecotype (Fig. 4a and b) and results for all taxa are given in Supplementary Data 1. We can broadly group the microbiota taxa into five categories based on their coefficient estimates: (i) significant in both classes with different directions; (ii) significant in both classes with the same direction; (iii) significant in prot-CARB only, (iv) significant in PROT-carb only and (v) not-significant in both classes. The first category “significant in both classes with different directions” represents consistent abundance changes in both nutritional-ecotypes (Fig. 4b). It was noted that the genera *Fusicatenibacter* and *Blautia* showed consistent negative coefficients in both PROT-carb and prot-CARB nutritional-ecotypes. Such genera may be considered stable PD microbial signatures, with several studies showing their underrepresentation in PD. [19, 20, 38–42]

The underrepresentation of *Fusicatenibacter* and *Blautia* was further validated using data from eight independent PD microbiome studies (Table 2). We processed the publicly available datasets using the dada2 pipeline [49](v1.16) and taxonomy reference “*silva* 138” [48, 50]. The relative abundance changes of the genus *Fusicatenibacter* were examined across all datasets, as shown in Fig. 4c. In all but one dataset [37], *Fusicatenibacter* had significantly lower relative abundance among PD individuals. Similar results were observed for *Blautia* (Supplementary Fig. 5), verifying NEMoE’s ability to identify consensus microbial signatures of PD in multiple independent cohorts.

**Table 2:** Summary of eight publicly available Parkinson’s disease microbiome studies used for validation of the NEMoE model

Study	Design	Country	Sample size	Sampling	DNA extraction	16S region	ENA Accession Number
Lubomski_0 [19, 39] Lubomski_6 Lubomski_12	Longitudinal	Australia	74PD, 74HC	Home collection, stored at −80 °C	MP Biomedicals FastDNATM SPIN Kit	V3-V4	PRJNA808166
Wallen_1 [36]	Cross-sectional	USA	323PD, 184HC	Home collection, swabs, stored at −20 °C	MoBio PowerSoil DNA Isolation Kit	V4	PRJNA601994
Wallen_2 [36, 43]	Cross-sectional	USA	197PD, 130HC	Swabs, delivered at RT	MoBio PowerMag Soil kit	V4	PRJNA601994
Aho (baseline) [44] Aho (follow-up)	Longitudinal	Finland	64PD, 64HC	Home collection, DNA stabilizer, stored in fridge	PSP-Spin Stool Kit	V3-V4	PRJEB27564
Weis [45]	Cross-sectional	Germany	34PD, 25HC	MED AUXIL fecal collector set	FastDNA Spin Kit	V4-V5	PRJEB30615
Pietrucci [46]	Cross-sectional	Italy	80PD, 72HC	Home collection, DNA stabilizer	PSP-Spin Stool Kit	V3-V4	PRJNA510730
Scheperjans [47]	Cross-sectional	Finland	72PD, 72HC	Home collection, DNA stabilizer, stored in fridge	PSP-Spin Stool Kit	V1-V3	PRJEB4927
Jin [48]	Cross-sectional	China	72PD, 68HC	NA	NA	V3-V4	PRJEB588834

Studies Lubomski_0, Lubomski_6, and Lubomski_12 were part of the same longitudinal data set by Lubomski and colleagues [2] and they represent samples that were measured at 0, 6, and 12 months, respectively

Studies Aho (baseline) and Aho (follow-up) were part of the same longitudinal data set by Aho and colleagues [44]. The same subjects were measured twice, at baseline and then later at follow-up, which was on average 2.25 years apart

Studies Wallen_1 and Wallen_2 were part of two large cohort studies set by Wallen and colleagues [38]

### Identification of the microbiome that are differentially represented in specific nutritional classes

We note that taxa categories (i)–(iii) represent differential abundance changes that are unique in the two nutritional-ecotypes prot-CARB and PROT-carb, which indicate some microbial signatures of PD are diet-specific (Fig. 4c). We discovered that the genus *Escherichia-Shigella* was significantly underrepresented in the prot-CARB nutritional-ecotype but not in the PROT-carb ecotype. This genus belongs to the family *Enterobacteriaceae* (including *E. coli*, *Shigella*, *Salmonella*, and *Klebsiella*), which are facultative anaerobes and known for utilizing soluble sugars as a carbon source. When an individual’s diet has a higher intake of sugars (or simple starch) it can be expected that the relative abundance of these microbiota will likely increase. Recent studies found that *Escherichia-Shigella* is a pathogenic bacteria that potentially reduces short-chain fatty acid production and produces endotoxins and neurotoxins [51, 52].

We also found a significant increase in the relative abundance of the genus *Akkermansia*, but only in the PROT-carb class (Fig. 4d). These bacteria are known to impact immune response and constipation, with many studies reporting an overrepresentation in PD [39, 40, 42, 53]. *Akkermansia* breaks down mucins and turns them into short-chain fatty acids; further, their relative abundance is thought to increase when “diet-specialize bacteria” decline as a direct impact of changes in microbially accessible carbohydrates (MAC). Generally, a low carbohydrate diet will lower MAC, thus lowering the number of diet-specialist microbes and allowing *Akkermansia* to become overrepresented, consistent with our discovery.

Most importantly, neither of these two genera (*Escherichia-Shigella*, *Akkermansia)* was discovered in our previous analysis using the ALDE model [54], where both classes were combined for microbiome biomarker identification (*Escherichia-Shigella: p*-value 0.14, *Akkermansia: p*-value 0.55) [20]. This highlights the relevance and importance of nutritional-ecotypes identification in microbiome marker discovery.

## Discussion

The aim of this study is to investigate and unravel the complex interaction between diet, the microbiome and an individual’s health. We achieve this by exploring the effects of dietary pattern (or composition) on the relationship between the microbiome and host health and by developing a method called NEMoE that detects such heterogeneity. Through a series of simulation studies, NEMoE shows strong prediction performance when the underlying data show heterogeneity explained by different nutrient intake. Furthermore, we illustrate the practical performance of NEMoE on a gut microbiome PD study in which nutritional-ecotypes and microbial signatures of disease are found. We show that NEMoE outperforms the predictive accuracy of previous models (higher AUC) and identifies multiple known PD microbiome markers. Two different nutritional-ecotypes are also identified within our data with distinct protein-to-carbohydrate intake ratios and novel candidate signatures that were indicative of a diet-specific cohort.

While we focus on the discovery of microbial signatures of PD by splitting the population based on dietary profile, the architecture of NEMoE means its flexible algorithm can take different types of data for subcohort detection (data used for gating networks) or biomarker identification (data used for expert networks). Therefore, an alternate research question could be to identify nutrients as disease markers for diverse microbiome profiles, and the NEMoE system can readily adapt to this new problem by changing the input of the gating network and experts network. Often, clinical knowledge or interest guides the decision on question formulation. However, if we consider both the dietary and microbiome profiles to be equivalent proxies for one’s nutrition system, then performing NEMoE in two different ways allows us to empirically compare the effectiveness of nutritional signatures versus microbial signatures and provides us with insight into the natural heterogeneity in the microbiome and in nutritional intake.

NEMoE is designed to partition samples based on their associated nutrient intake and can be viewed as a data-driven strategy for subcohort or latent class identification. An alternative option is to investigate a knowledge-driven strategy to achieve the same goal and one example is the use of “enterotype.” Similar to unsupervised learning, stratifying samples based on “enterotype” while providing an alternative way to stratify samples, does not explicitly take disease prediction performance into account. As a result, the aggregate predictive ability of the three separate enterotypes is lower than the nutritional-ecotypes division discovered by the NEMoE approach.

The proposed NEMoE method is based on diet-microbiome-host health interaction. However, it is not restricted to diet and microbiome data. Our method can be expanded to other multi-omics studies to identify subcohorts determined by the heterogeneity in relationships between covariates and response. One potential application is in the clinical heterogeneity of the relationship between multi-omics and host health. In such scenarios, the subcohorts are determined by their clinical index while the omics data are used to model the relationship between host health and information from a specific molecular platform.

In summary, we present NEMoE, a novel statistical method to model heterogeneity of diet and the gut microbiome in disease. NEMoE identifies nutritional-ecotypes based on a maximum likelihood framework and using an Expectation-Maximization step to estimate the model parameters. Our proposed framework also enables identification and then accounts for multiple levels of structure in the feature set, a unique characteristic in microbiome data, where we are able to estimate a shared latent class for each individual at different taxonomic levels. Effectiveness of NEMoE is validated at three levels. First, we demonstrate through a series of extensive simulation studies the model’s ability to accurately identify latent classes and to increase microbiome predictability. Second, we validate the performance of NEMoE on a real disease dataset and show that this method outperforms existing two-stage methods. Finally, the downstream impact and practical importance of NEMoE is further demonstrated by the discovery of diet-specific PD microbiome markers, such as *Escherichia-Shigella* and *Akkermansia*, which are not identified by the ALDE model [54].

## Methods

### Data collections

#### In-house studies

Our in-house gut microbiome PD data collection includes stool samples from 101 PD patients and 83 healthy controls across three timepoints (0-, 6-, and 12-month time points). The samples were collected and 16S rRNA V3–V4 amplicon sequencing was performed on an Illumina MiSeq platform. Details of the experimental setting can be found in Lubomski et al. [19, 20]. We denoted data corresponding to each timepoints as Lubomski_0, Lubomski_6, and Lubomski_12, respectively.

#### PD-diet

Dietary information was collected by a comprehensive Food Frequency Questionnaire and resulted in a table of nutrient intake with 23 macronutrients, presented earlier [43]. Details of the sample information and sequence processing can be found in Lubomski et al. [19, 20].

#### Public validation (PV) studies

We curated a series of datasets from eight different publicly-available microbiome studies [37, 38, 44–46, 51] to further validate results from NEMoE. All the datasets were processed using the dada2 pipeline [49] (v1.16) and microbiome taxa were annotated using taxonomy reference “silva 138” [48, 50]. Samples with low sequence reads (<1000) were excluded from the analysis. More information on these datasets can be found in Table 2. For the longitudinal datasets Aho [44], the data for baseline and follow-up, which were collected after 2.5 years, are denoted as Aho (baseline) and Aho (follow-up) respectively.

### Data processing

#### PD-microbiome data processing

We excluded 7 samples with extremely large energy intake (>20,000 kJ per day), one subject with low microbial read counts (total counts < 10,000), and two samples with missing nutrition measurements, resulting in 175 samples (75 HC individuals and 100 PD individuals). Raw counts from microbiome data were first normalized by total sum scaling, i.e., the counts (totals) were normalized into a composition proportion. Then core microbial features were kept and further transformed: Features that had more than 30% zeros in the *n* samples and features which had sample variance smaller than 10^−5^ were filtered out at each taxonomic rank resulting in the core microbial features of 7 Phylum, 19 Order, 27 Family, 41 Genus, and 101 ASVs, and 3,152,746 total reads were kept from 6,024,011 reads; variance stability transformation, *i.e.* an arcsin square root transformation, was performed on taxa proportion [47, 55]; the arcsin transformed data were further standardized to have mean zero and unit variance (z-score). We also performed z-score and central log transformation and the corresponding result are shown in Fig. S7.

#### PD-diet features construction

In addition to the nutrients intake values, we calculated the percentage of energy intake as protein (%EP), percentage of energy intake as fat (%EF), percentage of energy intake as carbohydrate (%EC), and protein intake and carbohydrate intake ratio (P:C) as additional variables. These transformations of nutritional features are widely used in nutri-omics studies [56, 57]. All of the 27 nutritional features were z-scored.

### Nutrition-ecotype mixture of expert (NEMoE) model

The development of NEMoE was inspired by a mixture of experts approach to model heterogeneous data as shown in Supplementary Fig. 2a. In machine learning, the concept of “*gate*” [58] can be thought of as a decision-making component given some input. Our approach consists of two key components, a “*gating network*” that is set up to determine which nutritional-class the sample belongs to and a “*k-experts network*” of size *k* to build classifiers for each nutritional-class. NEMoE uses a regularized MoE (RMoE) model, which adds elastic-net penalties to both the gating network and the experts network. Regularization is needed here because a non-regularized MoE does not extend to a large *p* small *n* framework [59] where the number of features (*p*) is much larger than the number of samples (*n*). This data characteristic often occurs in diet and microbiome data where there are many more microbial features (*p*) than individual samples (*n*). NEMoE further incorporates the taxonomic information into RMoE by jointly optimizing RMoE models from all taxonomic levels with the added constraint that all RMoE share the same gating network (Supplementary Fig. 2b).

#### Mathematical formulation of NEMoE

For a transformed microbiome data at taxonomic level *l,* we use the matrix ${X_{n\times {p}_l}}^{(l)}$to denote the relative abundance in *n* samples of *p*_*l*_ taxa. The corresponding diet information, measured as a nutrients intake matrix, is denoted as *W*_*n* × *q*_, where the *q* columns are the nutrient metrics for the same n samples Let *Y*_*n*_ denote the binary response of the health outcome, with *Y* = 1 and *Y* = 0 representing individuals with and without disease, respectively. NEMoE models the heterogeneous relationship between the microbiome and the health outcome by a mixture distribution, i.e.

1 $${P}_l\left(Y=1|{X}^{(l)},W\right)={\sum}_{k=1}^K{\pi}_k\frac{\mathit{\exp}\left({X}^{(l)}{\beta_k}^{(l)}\right)}{1+\mathit{\exp}\left({X}^{(l)}{\beta_k}^{(l)}\right)},$$

where ${\pi}_k=\frac{\mathit{\exp}\left(W{\gamma}_k\right)}{\sum_{i=1}^K\mathit{\exp}\left(W{\gamma}_i\right)}$ is the nutrition class mixing weight of shared components determined by nutrients intake, and where *γ*_*k*_ and *β*_*k*_ are the corresponding effect size for the gating network and the experts network, respectively, and *K* denotes the predetermined number of nutrition classes.

NEMoE estimates the regularized sum of all levels of the log-likelihood function in Equation (1), where the regularization term consists of elastic net penalties for both the gating network and the experts network:

2 $$rLL={\sum}_{l=1}^L{\sum}_{k=1}^K\left\{{\sum}_{i=1}^n\mathit{\log}\left[P\left({Y}_i|{X_i}^{(l)},{W}_i\right)\right]-\phi \left({\lambda_{1k}}^{(l)},{\alpha_{1k}}^{(l)},{\beta_k}^{(l)}\right)\right\}-\phi \left({\lambda}_2,{\alpha}_2,\gamma \right),$$

where $\phi \left(\lambda, \alpha, \beta \right)=\lambda \Big[\alpha \left|\beta \right|+\frac{1}{2}\left(1-\alpha \right){\left\Vert \beta \right\Vert}_2^2$] is the elastic net penalty function and *λ*_1*k*_^(*l*)^, *α*_1*k*_^(*l*)^, *λ*_2_, *α*_2_ are the corresponding parameters for penalties in the experts network and in the gating function.

The regularized LL can be maximized through a proximal Newton Expectation Maximization algorithm [59]. Details of the optimization procedure can be found in the reference manual of the NEMoE package https://sydneybiox.github.io/NEMoE .

### Performance evaluation

#### Comparison methods

Table 1 contains a summary of all methods used in the comparison study. We included the most commonly used methods in microbiome analysis as well as a naive two-stage approach. All of the comparisons were performed on simulation datasets and on in-house data on the Genus level.

#### Naive two-stage approach

The approach first clustered the nutrition data using unsupervised learning methods such as *k*-means. Then, based on the clustering result, samples in each cluster were used to build a classification model of microbiome and health state. The choice of classification models we used in our simulation includes sparse logistic regression (glmnet v4.1-2), support vector machine (e1071 v1.7-11), and random forest (randomForest v4.6-14).

#### Differential abundance

We compared differential relative abundance between PD and HC in all datasets. The comparison was based on a non-parametric bootstrapping procedure. We resampled the data with replacement, then calculated the difference of the average relative abundance between PD and HC. This procedure was repeated 10,000 times for each taxon and the 95% confidence interval of the differential relative abundance was calculated.

#### Simulation framework

Our simulation first generated independent data of 2*n* samples from the procedure described above, then the first *n* samples were used for training and another *n* samples were used to calculate the predicted accuracy. The details of parameter settings in each simulation are described in Table 3.

Implementation

**Table 3:** Summary of Simulation settings

Simulation Description	*n*	*p* ^c^	*q*	*η*	*c*_*e*_	*c*_*g*_	*K*	*ρ*
Evaluate the effect of *n*	(100, 200, 500, 1000)	50	30	0.1	2	2	2	0
Evaluate the effect of *η*	200	50	30	(0, 0.1, 0.3, 0.5)	2	2	2	0
Evaluate the effect of *p*	200	(30, 50, 80, 100)	30	0.1	2	2	2	0
Evaluate the effect of *q*	200	50	(30, 50, 80, 100)	0.1	2	2	2	0
Evaluate the effect of *ρ*	200	50	30	0.1	2	2	2	(0, 0.1, 0.3, 0.5)
Evaluate the effect of *K*^a^	(100, 200, 500, 1000)	50	30	(0, 0.1, 0.3, 0.5)	2	2	3	0
Evaluate the multi-level data^b^	500	100	30	0.1	2	2	2	0

^a^For the evaluation of the effect of K, the underlying simulation data is generated based on *K*=3, while the fitted NEMoE is based using K ranging from 2 to 4

^b^For the evaluation of the multi-level, we compare the adjusted rand index between NEMoE using all 5 levels of data (Phylum, Order, Family, Genus, and ASV) with NEMoE using only one level data

^c^Except the evaluation of multi-level, all evaluations were performed based on single-level data. For the multi-level data, the number of variables for Phylum, Order, Family, Genus, and ASV levels are 30, 50, 80, and 100, respectively

## Supplementary Information

**Additional file 1: Supplementary notes**. **Supplementary Fig. 1.** Illustration of NEMoE and two-stage model. **Supplementary Fig. 2.** Graphical model representation of NEMoE. **Supplementary Fig. 3.** Nutrition classes determined by *k*-means do not show an informative relationship between microbiome and PD. **Supplementary Fig 4.** Simulation results of NEMoE and other methods under different settings. **Supplementary Fig 5.** External validation of consensus taxa *Faecalibacterium* and *Blautia*. **Supplementary Fig 6.** Prediction performance of different types of input for NEMoE. **Supplementary Fig 7.** ROC curves for different standardization methods of microbiome composition data analysis.

## Data Availability

All the data used were published previously and the corresponding information is shown in Table 2. All processed datasets are incorporated in a R data package that is freely available from our GitHub repository at https://sydneybiox.github.io/PD16SData.
