# Supplementary material for: NEMoE: a nutrition aware regularized mixture of experts model to identify heterogeneous diet-microbiome-host health interactions
Source: Microbiome. 2023 Mar 15;11:51. doi: 10.1186/s40168-023-01475-4 (PMC10015776; doi:10.1186/s40168-023-01475-4)
Supplement: Supplementary file 2 — Additional file 1: Supplementary notes. Supplementary Fig. 1. Illustration of NEMoE and two-stage model. Supplementary Fig. 2. Graphical model representation of NEMoE. Supplementary Fig. 3. Nutrition classes determined by k-means do not show an informative relationship between microbiome and PD. Supplementary Fig 4. Simulation results of NEMoE and other methods under different settings. Supplementary Fig 5. External validation of consensus taxa Faecalibacterium and Blautia. Supplementary Fig 6. Prediction performance of different types of input for NEMoE. Supplementary Fig 7. ROC curves for different standardization methods of microbiome composition data analysis. [file 40168_2023_1475_MOESM1_ESM.docx]

**NEMoE: A nutrition aware regularized mixture of experts model to identify heterogeneous diet-microbiome-host health interactions**

Xiangnan Xu^1,2^, Michal Lubomski^4,5,6#^, Andrew J. Holmes^1,7#^, Carolyn M. Sue^4,5^, Ryan L. Davis^5^, Samuel Muller^2,3*^, Jean Y.H. Yang^1,2,8*^

^1^ Charles Perkins Centre, The University of Sydney. Camperdown, NSW, Sydney, Australia.

^2^ School of Mathematics and Statistics. The University of Sydney. Camperdown, NSW, Sydney, Australia.

^3^ Department of Mathematics and Statistics, Macquarie University, NSW 2109, Australia.

^4^ Department of Neurology, Royal North Shore Hospital, Northern Sydney Local Health District, St Leonards, NSW, Australia.

^5^ Department of Neurogenetics, Kolling Institute, Faculty of Medicine and Health, University of Sydney and Northern Sydney Local Health District, St Leonards, NSW, Australia.

^6^ The University of Notre Dame Australia, School of Medicine, Sydney, NSW, Australia.

^7^ School of Life and Environmental Sciences, University of Sydney. Camperdown, NSW, Sydney, Australia.

^8^ Laboratory of Data Discovery for Health Limited (D^2^4H), Science Park, Hong Kong SAR, China

**Key Words: Microbiome, Nutrition, Latent class, Mixture of Experts**

*** Co-last authors**

**# Equal contributors**

**SUPPLEMENTARY NOTES:**

**Simulation studies**

Our simulation is inspired and integrated from multiple simulation studies. These include the generation of the nutrition data based on a multivariate Gaussian distribution ^[1](https://paperpile.com/c/juuBWc/QlsQm)^ and a sparse multinomial regression model; the generation of the microbiome data using a zero-inflated latent Dirichlet allocation model (zinLDA) ^[2](https://paperpile.com/c/juuBWc/pTpBL)^ and constructing the health outcome using sparse logistic regression . The details of the simulation are described as follows.

The simulation of the nutritional data consists of two main components, the values of the nutritional measurements (*W*) and the underlying latent class which we refer to as nutritional-ecotype (*Z*).

Constructing the effect size between *W* and *Z*:

For a given number of nutritional features *q* and given the total number of latent classes *K*, we first simulate the effect size between *W* and *Z*, denoted by the matrix $\gamma_{q\times K}$ where each element $\gamma_{jk}$, $j=1,\ldots,q$ and $k=1,\ldots,K,$ represents the effect size of the $j$^th^ nutrition feature on the $k$^th^ latent class. We randomly select five nutrition features to have non-zero effect size and its value is either $c_{g}$ or $-c_{g}$ with equal probability, *i.e.*

$\gamma_{jk}=\left\{ \begin{aligned} {2\cdot c}_{g}(B_{jk}-\frac{1}{2}), j\in A_{k} \\ 0, j\notin A_{k} \end{aligned} \right.$ (1)

where $B_{jk}\sim Bernoulli(\frac{1}{2})$ and $A_{k}$ denotes a set of nutritional variables with non-zero effect size randomly drawn from the *q* nutritional variables, $c_{g}>0$ is a constant that controls the overall strength of the effect size and $B_{jk}$ is a Bernoulli random variable with probability parameter 1/2. This is designed such that the effect of a nutritional feature can randomly have a positive or negative value with equal probability. Selection of five nutrition features allows only a fraction of nutrition features to contribute to the ecotype.

Simulating nutritional values *W* and nutritional-ecotype *Z*:

The nutritional values are simulated with a *q*-variate Gaussian mixture distribution with *K* components. Given the *i*^th^ sample, we first generate its nutritional-ecotype $Z_{i}$ by randomly drawing an index from the set $\{1,\ldots,K\}$ indicating which latent class it belongs to. For $Z_{i}=k$, its corresponding nutrition data $W_{i}$ is generated from a normal distribution

$W_{i}|Z_{i}=k\sim N(\mu_{k},\Sigma_{k}),$ (2)

where $\mu_{k}$ and$\Sigma_{k}$ are the mean and covariance matrix of the *k* mixture component, respectively. In our simulation, we set$\Sigma_{k}$ to be a matrix with diagonal entries equal to 1 and off-diagonal entries equal to $\rho$, we further set $\mu_{k}$ to be proportional to the effect size of the $k$^th^ latent class $\gamma_{k}$ *i.e.*

$\mu_{k}=\eta\gamma_{k}$. (3)

The use of Gaussian mixture distributions for nutrition data is an extension of the simulation method by Chen and colleagues^[1](https://paperpile.com/c/juuBWc/QlsQm)^. The levels of heterogeneity between dietary patterns were controlled by different levels of $\eta.$ A larger $\eta$ represents a greater gap between the means of the components and thus, the larger that gap the easier it is to distinguish the nutritional ecotype $k$ (Fig. 3a). In our simulation, we define “none”, “weak”, “mild” and “strong” separation by setting the maximum absolute value $\eta$ to be 0, 0.1, 0.3, and 0.5, respectively.

Simulating microbiome data *X*: The generation of the microbiome data follows a zero-inflated latent Dirichlet allocation model (zinLDA)^[2](https://paperpile.com/c/juuBWc/pTpBL)^. This model simulates several typical characteristics of the microbiome data including over-dispersion, zero-inflation and high dimensionality. The distribution of the microbiome data *X* is given by,

$X\sim zinLDA(\pi_{m},a_{m},b_{m},\alpha_{m},K_{m},N_{m}),$ (4)

where $N_{m}$ is the total number of reads, $K_{m}$ is the number of microbiome subcommunities, $\alpha_{m}$ is the parameter in the Dirichlet distribution, $\pi_{m}$ relates to the generation of the subcommunity, $a_{m}$ and $b_{m}$ are the parameters in the zero-inflated generalized Dirichlet distribution related to the generation of counts within each subcommunity. Our simulation uses the default parameters as in zinLDA^[2](https://paperpile.com/c/juuBWc/pTpBL)^: $N_{m}$ was drawn from a discrete uniform distribution with a lower bound of 5,000 and an upper bound of 25,000, $\alpha_{m}=10$, $K_{m}$= 5, $\pi_{m}=0.4,a_{m}=0.05$ and $b_{m}=10,$ respectively. The microbial counts at ASV level were generated using the corresponding distribution of *p* variables.

For each simulation, we generate *n* samples of microbiome data at 5 taxonomic levels, where level 1 to level 5 correspond to Phylum, Order, Family, Genus and ASV, respectively and ($p_{1},\ldots,p_{5}$) = (10, 20, 50, 80, *p*) corresponds to the number of microbiome variables for each level respectively. The hierarchical structure of the taxonomic table is generated by randomly grouping microbial features from the higher neighbouring level *l*+1 into the $p_{l}$ group when going from the (*l*+1) level to the l level. We denote $ASV^{(l)}(j)$as the set of taxa at ASV level that is mapped to taxa *j* at level *l.* The simulated microbiome data of the *l* taxonomic table was thus hierarchically aggregated from the taxa from the (*l+*1) taxonomic table and the corresponding counts matrix is denoted as ${X^{(l)}}_{n\times p_{l}}.$

Simulating health outcome *Y*: The relationship between microbiome and health outcome was simulated based on a mixture sparse logistic regression which extends the model by Dong and colleagues ^[3](https://paperpile.com/c/juuBWc/Uwnx0)^. We simulate the binary health outcome $Y$in three steps.

Step 1: Simulate the microbial signatures of each latent class at ASV level. The microbial signatures were selected from candidates’ taxa that satisfy two conditions: prevalence is larger than 50% (counts are non-zero in at least 50% of samples) and the variance of its relative abundance is larger than $10^{-6}$. Then we randomly select 5 taxa from the candidates taxa for the *k*^th^ latent class and denote the corresponding sets of taxa as ${A_{k}}^{(ASV)}, k=1,...,K$.

Step 2: Simulate the effect size between microbiome $X^{(l)}$ and health outcome *Y* at the *l*^th^ taxonomic level and latent class *k*. We first generate the effect size at ASV level, which we denote as ${\beta_{k}}^{(ASV)}$. The *i*^th^ element of ${\beta_{k}}^{(ASV)}$ was generated as follows:

${\beta_{ik}}^{(ASV)}=\left\{ \begin{aligned} \frac{{2c}_{e}}{\sigma_{ik}}\left( B_{ik}-\frac{1}{2} \right), i\in{A_{k}}^{(ASV)} \\ 0, i\notin{A_{k}}^{(ASV)} \end{aligned} \right.,$ (5)

where $B_{ik}\sim Bernoulli\left( \frac{1}{2} \right), \sigma_{ik}$ is the standard deviation of taxa *i* in nutrition class *k* and $c_{e}>0$ is a constant that controls the strength of the effective size, and $B_{jk}$ is a Bernoulli random variable with probability parameter 1/2. Then the effect size of the *j*^th^ taxa at level *l* (${\beta_{jk}}^{(l)}$) is generated by aggregating its corresponding effect size at ASV level,

${\beta_{jk}}^{(l)}=\sum_{i\in{A_{k}}^{(ASV)}} {\beta_{ik}}^{(ASV)}$. (6)

Note that for taxonomic level *l*, the corresponding effect sizes were generated as the sum of the level *l+1* coefficients similar to as in Wang and colleagues ^[4](https://paperpile.com/c/juuBWc/UJCLg)^. Here, the strength of effect size is inversely proportional to $\sigma_{ik}$ and ensures that the contribution of the microbiome signature is not mainly affected by its relative abundance.

Step 3: Simulate the health outcome *Y*. We simulate the probability that “the health outcome *Y* equals 1” as an average of all 5 levels mixture sparse logistic regression with the mixing weight $\pi_{k}=\frac{exp(W\gamma_{k})}{\sum_{i=l}^{K} exp(W\gamma_{i})}$,

$P(Y=1|X^{(1)},\ldots,X^{(5)},{{W,\beta}_{k}}^{(1)},\ldots,{\beta_{k}}^{(5)},\gamma) =\frac{1}{5}\sum_{l=1}^{5} \sum_{k=1}^{K} \pi_{k}\frac{exp(X^{(l)}{\beta_{k}}^{(l)})}{1+exp(X^{(l)}{\beta_{k}}^{(l)})}$. (7)

For given microbiome data ${X^{(l)}}_{n\times p_{l}}$, nutrition data $W_{n\times q}$ and the corresponding effect size $\gamma$ and ${\beta_{k}}^{(l)},(l=1,\ldots,5$). we draw *n* Bernoulli pseudo-random samples with probability parameter given by equation (7) to obtain the binary health outcome vector $Y_{n\times1}$.

**REFERENCES**

1. [Chen, J. & Li, H. Variable Selection for Sparse Dirichlet-Multinomial Regression with an Application to Microbiome Analysis.](http://paperpile.com/b/juuBWc/QlsQm) *[Ann. Appl. Stat.](http://paperpile.com/b/juuBWc/QlsQm)***[7](http://paperpile.com/b/juuBWc/QlsQm)**[, (2013).](http://paperpile.com/b/juuBWc/QlsQm)

2. [Deek, R. A. & Li, H. A Zero-Inflated Latent Dirichlet Allocation Model for Microbiome Studies.](http://paperpile.com/b/juuBWc/pTpBL) *[Front. Genet.](http://paperpile.com/b/juuBWc/pTpBL)***[11](http://paperpile.com/b/juuBWc/pTpBL)**[, 602594 (2020).](http://paperpile.com/b/juuBWc/pTpBL)

3. [Dong, M., Li, L., Chen, M., Kusalik, A. & Xu, W. Predictive analysis methods for human microbiome data with application to Parkinson’s disease.](http://paperpile.com/b/juuBWc/Uwnx0) *[PLoS One](http://paperpile.com/b/juuBWc/Uwnx0)***[15](http://paperpile.com/b/juuBWc/Uwnx0)**[, e0237779 (2020).](http://paperpile.com/b/juuBWc/Uwnx0)

4. [Wang, T. & Zhao, H. Constructing Predictive Microbial Signatures at Multiple Taxonomic Levels.](http://paperpile.com/b/juuBWc/UJCLg) *[J. Am. Stat. Assoc.](http://paperpile.com/b/juuBWc/UJCLg)***[112](http://paperpile.com/b/juuBWc/UJCLg)** [1022–1031 (2017).](http://paperpile.com/b/juuBWc/UJCLg)

### SUPPLEMENTARY FIGURES

**Supplementary Fig. 1: Illustration of NEMoE and two-stage model. a**, A common workflow of a two-stage model: first clusters the cohort based on the nutrition intake, then builds a model between microbiome data and health outcome within each cohort. **b**, Illustration of a two-stage model with two latent classes. **c**, Illustration of two methods NEMoE and naive two-stage model in both nutrition space and microbiome space. Naive two-stage model identified two latent classes showed best separation in the nutrition space but do not count for the relationship between microbiome and health outcome; two latent classes identified by NEMoE showed differential relationship between microbial features and health outcome.

**Supplementary Fig. 2: Graphical model representation of NEMoE. a**, A graphical model representation of the mixture of experts model. **b**, A graphical model representation of the NEMoE extends the MoE to multi-level with a shared gating network.

**Supplementary Fig. 3: Nutrition classes determined by *k*-means do not show an informative relationship between microbiome and PD. a**, PCA plot of scaled nutrient intake for subjects colored by two latent classes estimated by *k*-means. **b**, Loadings of the first two PCs. Loadings of the second PC shared some important variables with the coefficients of the gating network in NEMoE. **c**,**d**, Variables selected by NEMoE do not show a clear difference between PD and HC.

**Supplementary Fig 4: Simulation results of NEMoE and other methods under different settings. a**, simulation results under different simulation parameters settings including the separation parameter $\eta$, sample size *n*, number of variables in the gating network *q* and in the experts network *p* and correlation $\rho$ between variables. The NEMoE achieves best predictive performance compared with others in all settings with latent class structure. **b**, Simulation results of three latent classes with different *n* and $\eta$. NEMoE III and NEMoE IV perform well under most parameter settings. **c**, Time consumption of different EM-algorithms implemented in NEMoE. CEM was the fastest while achieving lower regularized LL, while EM and SAEM achieved higher regularized LL but required more time. **d**, Comparison between different taxa levels with NEMoE in estimation of shared latent classes. Level *K* represents fitting RMoE with data in level *K* (*K*=1,2,3,4). The ARI is calculated by comparing the estimated latent class and the generated true latent class.

**Supplementary Fig 5: External validation of consensus taxa *Faecalibacterium* and *Blautia*. a**, Validation of differential relative abundance of genus *Faecalibacterium* in eight different datasets. **b**, Forest plot showing the validation of differential relative abundance of genus *Blautia* in eight different datasets.

**Supplementary Fig 6: Prediction performance of different types of input for NEMoE**. Using nutrients intake as the input of the gating network and microbiome as the input of the experts network showed better prediction performance than for the other cases. NEMoE with two nutrition classes showed best prediction performance in our dataset.

**Supplementary Fig 7: ROC curves for different standardization methods of microbiome composition data analysis.** ‘Scale’(zscore), ‘clr’ (central log ratio transformation ) and ‘asin’ (arcsine transformation ) denote the three methods assessed for transformation of microbiome data. The LOOCV-AUC for NEMoE standardized with scale, clr and asin transformations are 0.74, 0.76 and 0.78 respectively.
